# Simultaneous Left and Right Atrial Appendage Thrombi on Cardiac Computed Tomography

**DOI:** 10.3390/reports9020160

**Published:** 2026-05-19

**Authors:** Mustafa Mohamed, Guillaume Fahrni

**Affiliations:** Department of Diagnostic and Interventional Radiology, Lausanne University Hospital, University of Lausanne, 1011 Lausanne, Switzerland

**Keywords:** cardiac computed tomography, atrial appendage, left atrial appendage, right atrial appendage, thrombus, atrial fibrillation

## Abstract

We report an unusual case of simultaneous left and right atrial appendage thrombosis identified on contrast-enhanced cardiac computed tomography angiography (CT) during pre-procedural evaluation in a patient with permanent atrial fibrillation and structural heart disease. Cardiac CT demonstrated well-defined filling defects within both atrial appendages on arterial and delayed phases, consistent with intracavitary thrombi. The patient was already receiving long-term oral anticoagulation for atrial fibrillation. In this case, antithrombotic management was not modified after multidisciplinary clinical assessment, as the patient remained asymptomatic and at high bleeding risk. This case highlights the diagnostic value of multiphasic cardiac CT in pre-procedural imaging, and underscores that systematic bilateral appendage assessment is essential, as right atrial appendage thrombus may otherwise go undetected.

A 78-year-old man with permanent atrial fibrillation (AF), severe calcific aortic stenosis (valve area 0.75 cm^2^; mean gradient 41 mmHg), and extensive cardiovascular comorbidities, including insulin-treated type 2 diabetes and multivessel coronary artery disease, underwent contrast-enhanced cardiac CT as part of a pre-procedural assessment for transcatheter aortic valve implantation (TAVI). Recent transthoracic echocardiography had confirmed a left ventricular ejection fraction of 57–64% and severe biatrial enlargement, but was limited in its assessment of the atrial appendages. The patient’s profile was notable for a high thromboembolic risk (CHA_2_DS_2_-VASc score of 6) and a high bleeding risk (HAS-BLED score of 4). The examination was performed on a 256-slice CT (Revolution CT, GE Healthcare) using a multiphasic acquisition protocol with prospective ECG-gating. A 70 mL bolus of contrast medium was injected at 4.0 mL/s, followed by a saline flush. This protocol included an initial full-cycle arterial phase followed by a narrow-peak delayed phase acquired 100 s later, allowing for reliable differentiation between true thrombus and slow-flow phenomena. Reconstructions were performed at a slice thickness of 1.5 mm. Persistent low-attenuation structures within the appendages on delayed imaging (mean density 50 ± 15 HU) confirmed the presence of concomitant thrombi in both atrial appendages, a rare finding (Figure 1). There was no evidence of embolic complications. The diagnosis was established based on the high specificity of persistent filling defects on delayed-phase CT.

The patient was already receiving therapeutic oral anticoagulation with rivaroxaban, which was maintained at a dose of 15 mg daily during hospitalization due to impaired renal function (eGFR 30 mL/min/1.73 m^2^). Following a multidisciplinary discussion, no treatment escalation (such as switching to a different anticoagulant or adding an antiplatelet agent) was deemed necessary, as the patient was hemodynamically stable, asymptomatic, had no evidence of systemic or pulmonary embolism, and remained at high bleeding risk [1]. Furthermore, invasive strategies like left atrial appendage occlusion were not considered appropriate in this acute thrombotic setting.

Atrial appendage thrombus forms in the setting of blood stasis, endothelial dysfunction, and hypercoagulability, with AF as the dominant clinical setting because it promotes impaired appendage emptying and spontaneous echo contrast. Thrombus is far more common in the left atrial appendage and is widely recognized as the principal source of AF-related thromboembolism; reported detection rates vary across cohorts, from about 1.2% to 22.6% in reviews and 4% to 19% in selected TEE populations [2]. By contrast, right atrial appendage thrombus is distinctly uncommon; in one prospective TEE study, it was found in only 0.73% of patients versus 9.3% in the left appendage [3]. Truly simultaneous bilateral appendage thrombosis remains an exceptional clinical entity, with only a few cases reported in the literature [4,5,6]. While left-sided thrombi are the primary source of systemic embolism, the coexistence of RAA thrombus introduces a potential dual embolic risk, including the possibility of silent or symptomatic pulmonary embolism, which may be overlooked in routine AF management [7]. As highlighted by Davila et al., the lower detection rate of RAA thrombus compared to that of the LAA (90% vs. <2%) might be partly due to anatomical differences, such as the wider neck and different remodeling patterns of the right appendage, as well as more challenging visualization on routine TEE. When present, right-sided thrombus is usually associated with the same prothrombotic substrate, AF or flutter, low appendage flow velocities, and spontaneous echo contrast. Because anticoagulation does not completely eliminate appendage thrombus, thrombus can still occur despite therapeutic treatment, especially in patients with advanced structural heart disease and multiple comorbidities. From a clinical perspective, this case underscores the importance of systematically evaluating both appendages during pre-procedural cardiac CT, as the presence of a right-sided thrombus, often overlooked, may have implications for the potential risk of pulmonary embolism or influence the planning of right-heart interventions.

Echocardiography, especially TEE, remains the reference test for intracardiac thrombus, and spontaneous echo contrast is a classic sign of blood stasis on ultrasound [8]. TEE is recommended in current AF management guidelines as the reference standard for exclusion of left atrial appendage thrombus prior to cardioversion or structural heart interventions [9]; however, TEE is semi-invasive and may have limitations in complete visualization of both atrial appendages, particularly the right atrial appendage. In this context, cardiac CT provides a non-invasive alternative with high diagnostic accuracy [10,11]. On CT, the key sign is a persistent low-attenuation filling defect that remains visible on delayed imaging rather than disappearing with contrast washout; delayed-phase imaging improves confidence by separating true thrombus from slow-flow [12]. Cardiac MRI adds specificity because thrombus is avascular, so it is identified by its characteristic tissue behavior on contrast-enhanced sequences, particularly delayed enhancement [13]. In practice, CT and MRI are complementary to ultrasound when echo is equivocal or when tissue characterization is needed. In the present case, although TEE and cardiac MRI could have provided complementary data, they were not performed in this clinical setting as the CT findings provided a high level of diagnostic confidence, and additional imaging was not expected to alter immediate management.

## Figures and Tables

**Figure 1:**
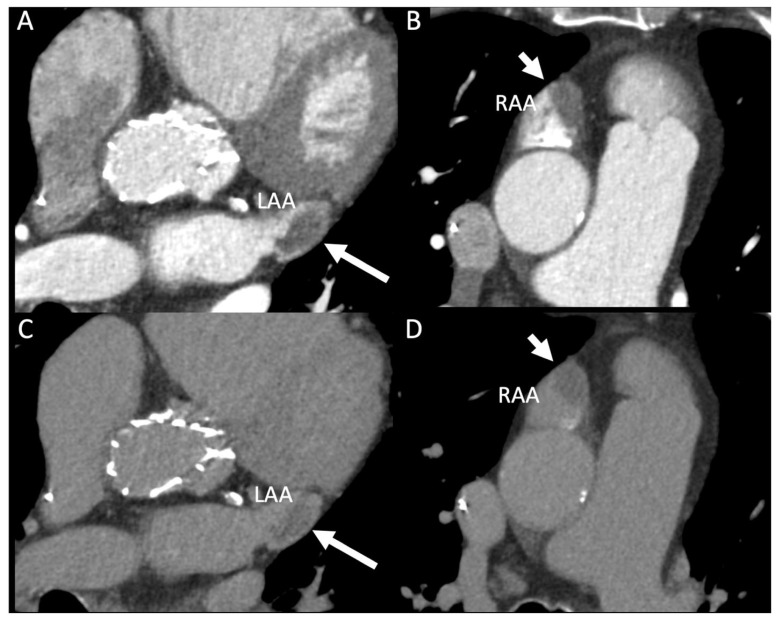
Contrast-enhanced cardiac computed tomography (CT) demonstrating simultaneous thrombi in the left and right atrial appendages. (**A**) Arterial-phase axial image showing a well-demarcated, low-attenuation filling defect within the left atrial appendage (long white arrow), compatible with thrombus. (**B**) Arterial-phase axial image demonstrating a similar filling defect within the right atrial appendage (short white arrow). (**C**) Delayed-phase image confirming persistence of the left atrial appendage filling defect without contrast enhancement (long white arrow), supporting the diagnosis of thrombus rather than slow-flow. (**D**) Delayed-phase image showing persistent non-enhancing material within the right atrial appendage (short white arrow), also consistent with thrombus.

## Data Availability

No new data were created or analyzed in this study.

## Abbreviations

The following abbreviations are used in this manuscript:

CT	Computed Tomography
AF	Atrial Fibrillation
TAVI	Transcatheter Aortic Valve Implantation
TEE	Transesophageal Echocardiography
LAA	Left Atrial Appendage
RAA	Right Atrial Appendage
HU	Hounsfield Units
eGFR	Estimated Glomerular Filtration Rate
MRI	Magnetic Resonance Imaging
